# Sleep Quality and Its Associated Factors among Low-Income Adults in a Rural Area of China: A Population-Based Study

**DOI:** 10.3390/ijerph15092055

**Published:** 2018-09-19

**Authors:** Wenwen Wu, Wenru Wang, Zhuangzhuang Dong, Yaofei Xie, Yaohua Gu, Yuting Zhang, Mengying Li, Xiaodong Tan

**Affiliations:** 1 Department of Occupational and Environmental Health, School of Health Sciences, Wuhan University; Wuhan 430071, China; wuwenwen108@126.com (W.W.); 13129957872@163.com (Z.D.); hh15629086961@163.com (Y.X.); yaohuawhu@163.com (Y.G.); yutingwhu666@163.com (Y.Z.); limengying1028@163.com (M.L.); 2 Alice Lee Centre for Nursing Studies, Yong Loo Lin School of Medicine, National University of Singapore, Singapore 117597, Singapore; nurww@nus.edu.sg

**Keywords:** China, rural area, low-income, sleep quality

## Abstract

Background: There is limited population-based research focusing on sleep quality among low-income Chinese adults in rural areas. This study aimed to assess sleep quality among low-income adults in a rural area in China and identify the association between sleep quality and sociodemographic, lifestyle and health-related factors. Methods: The study was conducted from September to November in 2017 using a cross-sectional survey questionnaire. A total of 6905 participants were recruited via multistage, stratified cluster sampling. Data were collected using the Chinese versions of Pittsburgh Sleep Quality Index and Food Frequency Questionnaire, while we also determined the sociodemographic profiles of the participants. Results: The mean age of the sample was 58.71 ± 14.50 years, with 59.7% being male, while the mean duration of daily sleep was 5.95 ± 1.31 h, with 56.7% reportedly experiencing poor sleep quality. Multiple regression analysis revealed that older age, unemployment, lower income, disability and chronic disease comorbidities were significant factors associated with an increased risk of poor sleep quality for both genders. Moreover, married and higher education level were associated with decreased risk of poor sleep quality for females, while a meat-heavy diet and illness during the past two weeks increased the risk of poor sleep quality for males. Conclusions: Sociodemographic, lifestyle and health-related factors had an impact on the frequently poor sleep quality of low-income Chinese adults in rural areas. Thus, comprehensive measures must be developed to address the modifiable predictive factors that can possibly enhance sleep quality.

## 1. Introduction

Sleep is an important determinant of health because it is closely associated with mortality [1] and morbidity [2]. Poor sleep quality can lead to poor concentration and memory [3] as well as slow responses. According to the rural poverty standard of 2300 RMB [4] annual income per person of a household defined by the Chinese government, approximately 43.35 million Chinese people can be classified as belonging to the low-income population. This number accounts for 4.5% of the entire population according to the 2016 Statistical Communique on China’s national economic and social development [5]. In Hong Kong [6], 39.4% of adults reportedly experience poor sleep quality compared with 13.1% in Korea [7], 32.1% in Austria [8] and 16% in mainland China [9]. Nevertheless, population-based prevalence data on sleep quality among low-income Chinese adults in rural areas are limited.

Demographic factors, such as age, gender, education level, marital status, employment and living alone [3,9,10,11,12], have been linked to sleep quality. Other studies have also found that smoking has a dose-response relationship with sleep. Thus, higher tobacco consumption results in shorter sleep duration [13]. People who abstain from drinking alcohol have also been found to be more sleepless than those who drink moderate amounts [13]. Other factors, such as dietary habit, illness during the past two weeks, disability and physical diseases, have also been associated with poor sleep quality [13,14]. The majority of the previous studies on sleep quality and its related influencing factors were conducted in developed countries. However, cultural factors play an important role in sleep-related disturbances [15]. Thus, the findings obtained from Western countries may not be applicable to countries in the East due to their different social and cultural backgrounds. A few studies on the prevalence and associated factors of poor sleep quality [3,16] among the general Chinese population have been conducted, but low-income adults in rural areas have been overlooked. Moreover, previous studies failed to include other factors, thus leading to incomplete results and lack of credibility.

China’s rapid economic growth in recent years has led to various social problems, such as a large number of young people leaving rural areas, widening gap between the rich and the poor and rising divorce rates [17,18]. The effects of these changes on sleep quality in rural areas must be verified.

Thus, this study is designed to evaluate the prevalence and distribution of sleep quality as well as identifying the sociodemographic, lifestyle and health-related factors that affect it.

## 2. Materials and Methods

### 2.1. Participants

China is currently implementing a nationwide, targeted poverty alleviation program to eliminate poverty in rural areas. The government has implemented a strict registration system [4] for all low-income villagers to accurately identify the benefactors of this program. Ezhou City in central China, which consists of three counties, 25 townships and 286 villages, has a total population of 1.0685 million, 5.5% of which belongs to the low-income population of the rural area according to the data released by the Ezhou Bureau of Statistics in 2016. Our sample is mainly comprised of low-income adults (age ≥ 18 years) from the rural area of Ezhou City, who were identified by the group that is supervising the provincial poverty alleviation project.

A population-based, cross-sectional study was conducted from September 2017 to November 2017, while a multistage, systematic cluster sampling method was used to recruit the participants. Four towns were randomly selected from each county and three low-income villages were chosen from each town. A total of 7107 individuals were then randomly selected using the poverty registration system. After the exclusion of 8 individuals with psychiatric disorders, 12 with hearing impairment and dyslexia, 5 with dementia and 167 who refused to participate, the final sample was reduced to 6905 participants (with a response rate of 97.6%). The current cross-sectional data were collected entirely through questionnaires during face-to-face interviews in the homes of the participants as approved by the Research Ethics Board of Wuhan University Health Science Center (project identification code: JKGW20170202), which was carried out in accordance with the Helsinki Declaration of 1975. Moreover, written informed consent was obtained from each participant prior to the questionnaire.

### 2.2. Procedures and Measures

Before data collection, eight investigators participated in workshops conducted by Wuhan University. The investigators explained the objectives and procedures of the study to potential participants through a telephone conversation. Upon obtaining the participants’ verbal consent, the investigators used the questionnaires to conduct face-to-face interviews in the participants’ homes.

A set of questionnaires on sociodemographic, lifestyle and health-related factors that are associated with sleep quality was used for data collection.

The sociodemographic and lifestyle data used as exploratory variables included gender, age, marital status, employment, average household income, educational level and living arrangements.

The lifestyle factors included are smoking and alcohol status (response to “yes” or “no”), salt intake (g/d), oil intake (g/d) and dietary content. Salt mainly comes from condiments, such as table salt, sodium glutamate and soy sauce. We used a validated Food Frequency Questionnaire (FFQ) [19] to collect information on salt and edible oil intake, dietary content and number of household members who consumed meals at home over the past month. First, we calculated the monthly salt intake of each household. Individual salt and edible oil intakes were calculated by dividing the number of household members by the salt and edible oil intake of the entire household per month. The level of salt intake was categorized into four groups (≤6, >6 and ≤12, >12 and ≤18, >18), while edible oil intake was also categorized into four groups (≤25, >25 and ≤35, >35 and ≤45, >45). Meanwhile, dietary content was categorized into balanced, primarily vegetarian or primarily meat diets.

Health-related factors mainly included illness during the past two weeks, number of chronic diseases and self-reported disability. Illness during the past two weeks was defined as a sickness lasting for the past two weeks.

The number of chronic diseases was assessed via the participant’s response (“yes” or “no”) when asked if he/she has been diagnosed with hypertension, diabetes, migraine, asthma, thyroid disease, heart disease, thrombosis, bronchitis/emphysema, osteoporosis, arthritis, cancer, stomach/peptic ulcer, cerebrovascular disease and other major physical diseases. This assessment is similar to what Scott described in a previous study [20].

Self-reported disability was assessed using four questions adopted from a previous study [21]. The participants were asked if they had any of the following permanent conditions: (1) vision or hearing limitations, such as blindness or severe vision impairment and deafness or severe hearing impairment; (2) functional limitations or substantial restriction from basic physical activities, such as walking, climbing stairs, reaching, lifting or carrying; (3) physical conditions that lasted for at least six months, resulting in difficulty remembering or concentrating; and (4) limitations in daily activities, such as dressing, bathing or getting around inside the home.

Subjective sleep quality was assessed via the Pittsburgh Sleep Quality Index (PSQI), which was developed by Buysse et al. in 1989 [22]. Thus, far, this scale is the most comprehensive and widely used sleep quality questionnaire. Moreover, previous studies [23] have proven its sensitivity, accuracy, comprehensibility and reproducibility. Tsai et al. [24] proved the reliability of the Chinese version of the Pittsburgh Sleep Quality Index (CPSQI), which was consistent with the present study that had a Cronbach’s alpha of 0.72. The CPSQI scale consists of 19 items that evaluate the sleep status in the previous month from multiple perspectives [13]. All items generate seven clinically derived sleep quality components, which are namely subjective quality, latency, duration, habitual efficiency, disturbances, use of medications and daytime dysfunction. The sum of these seven components is the global score of the CPSQI scale (range of 0–21), with lower scores indicating better sleep quality. Good and poor sleepers are distinguished via the diagnostic sensitivity and specificity of a total CPSQI score >5 (89.6% and 86.5%, respectively [3]). According to Buysse’s research [22], a total score of more than 5 has the best sensitivity and specificity for classifying poor sleep quality. In this study, a total CPSQI score of ≤5 was considered as good sleep quality, while a score of >5 was regarded as poor sleep quality.

### 2.3. Statistical Analysis

The statistical software Stata version 10.0 (Stata Corporation, College Station, TX, USA) was used to calculate the overall percentage of poor and good sleepers among low-income adults in the rural areas of China. The descriptive statistics of mean and standard deviation (SD) were used for continuous variables, while frequency and percentages were used for categorical variables. We examined the binary association between sleep quality (categorical variable) and another categorical variable using the Chi-squared test. The odds ratio (OR) and 95% confidence interval (95% CI) of the association between different factors and sleep quality were analyzed via univariable logistic regression analysis. Ultimately, the association of sociodemographic, lifestyle and health-related factors with sleep quality were identified through multiple logistic regression. Statistical significance was set at *p* < 0.05 and all of the *p* values were two-sided.

## 3. Results

### 3.1. Characteristics of Participants and Impact of Differences on Sleep Quality

Of the 6905 participants, 59.7% were male. The average age of the participants was 58.71 years (SD = 14.50), while more than half of them (*n* = 3608, 52.3%) were aged 60 years or older. Furthermore, 21.2% and 13.9% of the participants were regular smokers and current alcohol drinkers, respectively. More than half (*n* = 4135, 59.9%) were married, while 22.8% were living alone. The majority were unemployed (*n* = 6077, 88.0%), only received primary education or below (74.2%), living with others (77.2%) and with an average annual income per person of a household ≤1000 RMB (51.9%). In addition, 50.6% of the participants were found to have a salt intake of >6 and ≤12 g/d, while 52.3% have an oil intake of >25 and ≤35 g/d. In terms of illness and disabilities, 54.6% of the participants had an illness during the past two weeks, 29.9% reported a disability and 37.0% suffered from one or more disease(s). Of the 6905 subjects, 2990 (43.3%) were classified as good sleepers, while the remaining 3915 (56.7%) were classified as poor sleepers (male: 53.4% and female: 61.5%; *p* < 0.01). The results of the univariate analysis are shown in Table 1 and Table 2. For both males and females, the quality of sleep is associated with age category, marital status, employment, average annual income per person of a household, educational level, salt intake, oil intake, living arrangement, dietary content of food, disability and chronic diseases. Besides, poor sleep quality in females is also associated with the illness during the past two weeks.

### 3.2. Description of Sleep Quality and Its Components

**Figure 1:**
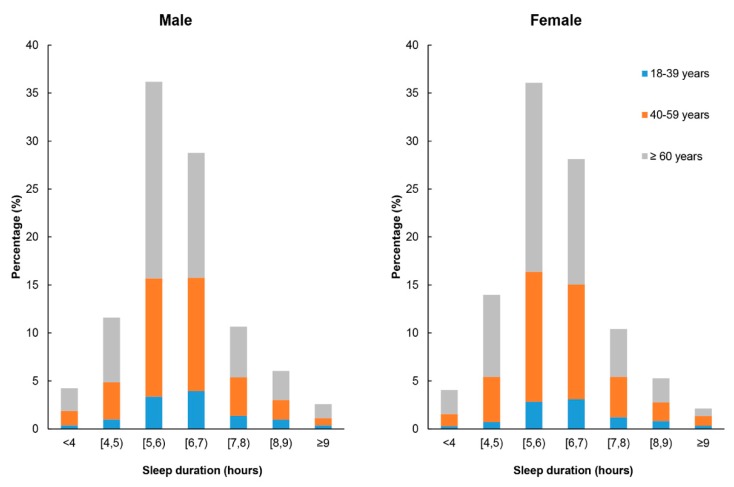
Distribution of sleep duration by age and sex. [4, 5): ≥4 and <5; [5, 6): ≥5 and <6; [6, 7): ≥6 and <7; and [7, 8): ≥7 and <8.

The participants have an average sleeping time of 6.60 ± 1.23 h. On average, they go to bed at 11:26 PM and get up at 6:03 AM. Figure 1 illustrates the distribution of sleep duration by age group and sex. The mean night sleep duration is 5.95 ± 1.31 h (5.99 ± 1.32 h in males, 5.75 ± 1.29 h in females; 6.24 ± 1.33 h in 18–39 years old, 6.00 ± 1.28 h in 40–59 years old and 5.85 ± 1.31 h in 60 years old or older). The participants’ average sleep latency was 39.07 min (SD = 25.76), while 35.3% were unable to fall asleep within 30 min. Meanwhile, 18.8% of the participants reported that they have more than 7 h of sleep per night. A total of 88.5% had a high sleep efficiency of over 85%, while 12.4% have used sleeping medication at least once in the previous month.

**Figure 2:**
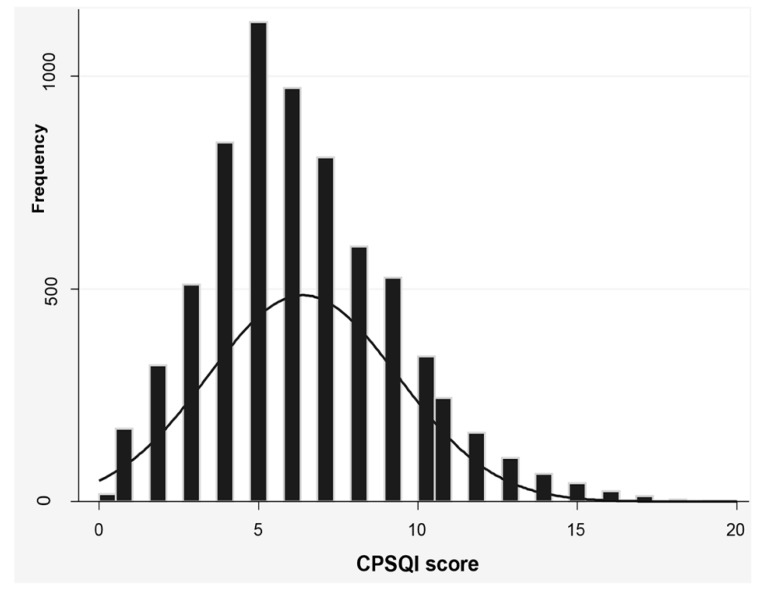
Histogram of CPSQI scores of the study sample (*n* = 6905). CPSQI: Chinese version Pittsburgh Sleep Quality Index.

As shown in Figure 2, most of the participants scored a CPSQI of 5–7 points, with a mean score of 6.39 ± 2.99. At the same time, the mean scores for self-rated sleep quality, latency, disturbance and daytime dysfunction were 1.29 (SD = 0.01), 1.28 (SD = 0.01), 1.42 (SD = 0.01) and 1.27 (SD = 0.01), respectively (Table 3). We evaluated the frequency distribution of CPSQI scores by gender and age. Each group exhibited significant statistical differences in terms of sleep quality. Table 3 shows that males scored significantly better on self-rated sleep quality (*p* < 0.001), latency (*p* < 0.001), efficiency (*p* < 0.001), disturbance (*p* < 0.001) and daytime dysfunction (*p* < 0.005). No significant difference was observed in sleep duration and need for sleep medications between males and females. Participants aged ≥60 years were more reluctant to obtain sleep medication compared with the other two age groups (*p* < 0.001). The older group also presented poorer self-rated sleep quality (*p* < 0.001), longer latency (*p* < 0.001), shorter duration (*p* < 0.001), lower efficiency (*p* < 0.001), more serious disturbance (*p* < 0.001) and daytime dysfunction (*p* < 0.001) compared to the younger group.

### 3.3. Determinants of Sleep Quality

Significant variables were included in the bivariate analysis for the final logistic regression model. As presented in Table 4 and Table 5, after adjusting for the age category, marital status, employment, average household income, living arrangement, educational level, salt intake, oil intake, dietary content of food, illness during the past two weeks, total number of chronic diseases and self-reported disability, we found that the age group was not a significant factor in determining sleep quality, except for participants aged 18–39 years (OR_total_: 0.46; and OR_male_: 0.51; and OR_female_: 0.41). Married people had 29 percent lower odds of poor sleep than single people (OR_total_: 0.71). In terms of gender, only female married individuals had 49% lower odds of experiencing poor sleep quality than divorced individuals/widows/widowers (OR_female_: 0.51). For both males and females, respondents who were employed had lower odds of reporting poor sleep quality (OR_total_: 0.49; and OR_male_: 0.49; and OR_female_: 0.48) compared with those who were unemployed. Participants with 1001–2300 RMB household annual income per person had 95% lower odds of experiencing poor sleep quality (1001–2300 RMB: OR_total_, 0.05; and OR_male_, 0.05; and OR_female_, 0.06). The higher the education level, the lower the odds of poor sleep quality (illiterate: OR_total_, 1.51; elementary school: OR_total_, 1.45; and junior middle school: OR_total_, 1.39). A similar association was found only in females (illiterate: OR_female_, 2.15; elementary school: OR_female_, 1.93). Living arrangement was significant in association with poor sleep quality in unadjusted analysis, but the association attenuated to non-significance in the adjusted analysis. The probability of poor sleep quality in both genders decreased in those that were not physically disabled and with no more than one chronic disease. In addition, male participants who had a balanced or primarily vegetarian diet and had not been sick during the past two weeks were statistically less likely to have poor sleep quality.

## 4. Discussion

In this study, we explored the prevalence and associated factors of poor sleep quality among low-income adults in a rural area of China. Most of the participants were elderly because many young people prefer to work in cities. Only the elderly and children remain in villages. The average sleep duration of our sample was lower than what was found previously among elderly adults in China [25] but similar to that of Hong Kong [26]. Insufficient sleep duration is common in modern society [27] where rural low-income residents have more outdoor work and life pressure.

The mean of the CPSQI score for the entire sample was categorized with poor sleep quality, which comprised more than half of the respondents. Staying up late and sleep latency were likely the main factors influencing the total CPSQI scores. The prevalence (56.7%) of poor sleep quality is much higher than the rate reported in prior studies that involved the rural older [28] and general adult population in mainland China [13], general population in Hong Kong [6], urban adults in Germany [29], workers in Brazil [30] and general Japanese population [31]. Meanwhile, Chiu et al. [32] found that 75% of the elderly reported poor sleep quality in Hong Kong, while Lo et al. [26] also reported a 78% prevalence of poor sleepers among Chinese seniors in general. The use of different samples may be the reason for this difference.

Females were more likely to have poor sleep quality than males, which is similar to the result of a previous study conducted in Hong Kong [26]. Gu et al. also reported that males generally experience good sleep quality [33]. This discrepancy may be due to various reasons, such as the level of female hormone secretion and different responses to stress as identified by economic status. Furthermore, in this study, more female were illiterate (49.4% vs. 23.8%), while more female also have chronic diseases (39.4% vs. 35.4%). These findings are consistent with a study conducted in Hong Kong [6], which indicated that socioeconomic factors and chronic diseases, rather than gender, may lead to a significant association between female individuals and poor sleep quality. Moreover, the sleep quality measured by the CPSQI of this study are based on subjective reports of the subjects instead of objective sleep quality. One study has shown that there is considerable consistency between subjective and objective sleep quality for males but not for females [34]. Another possible important reason may be that Chinese women may take more responsibilities in taking care of the family, especially in rural areas.

We also found that single female participants were more likely to have poor sleep quality compared with the married ones. Arber’s research also showed that previously married (whether divorced or widowers) women and men had a higher risk of poor sleep quality compared with their married counterparts, which can be explained by their more disadvantaged socio-economic status [35]. In this study, this association was found in females, but not in males. This may be because there is little difference in socioeconomic status between single and married men with low incomes. After controlling for confounding factors, such as gender, health-related and other sociodemographic factors, single participants were also more likely to have poor sleep quality compared with the married ones. However, Haselimashhadi et al. found that marital status does not affect the sleep quality of the elderly in Beijing [9]. Maybe this is because our subjects are not only elderly people, but also young people and middle-aged people.

Consistent with previous reports [36,37,38], our study indicated that lower education level results in a greater risk of poor sleep quality of all participants. A lower education level results in unhealthier lifestyle choices, resulting in decreased sleep quality. Moreover, education is associated with occupation and income level [39]. However, the logistic regression analysis of samples of different genders showed that this association was observed only in females. We speculate that this may be due to the fact that educational levels have a greater impact on women’s health-related cognitive abilities than male. In the present study, nearly 90% of low-income adults with a low education level were unemployed. Our findings show that having the lowest level of average annual income per person of a household (<1000 RMB) and unemployment also increase the risk of poor sleep quality for both males and females. A cross-sectional study in Philadelphia showed that people below the poverty threshold or unemployed individuals are more likely to be poor sleepers [12]. A lower income places greater pressure on an individual in terms of survival. This may be due to some negative emotions caused by survival pressure, such as anxiety.

Age also plays a role in determining sleep quality as young people are reported to have better sleep quality than middle-aged adults and elderly people in both genders [40]. As people age, they experience a decrease in the secretion of growth hormones related to deep sleep [41]. Researchers found that growth hormone levels in the body drop by 14% every decade from age 20 to 59. As people reach the age of 60 and beyond, the levels of growth hormone secretion decrease further [42].

In this study, no association between smoking and sleep quality was found among males and females. Previous studies have reported that smoking increases the risk of poor sleep quality [43,44]. However, another study have shown that smoking is only associated with sleep duration and not with overall sleep quality [45]. Moreover, we found no statistically significant difference in sleep quality between drinkers and non-drinkers. However, a study conducted by Jackson et al. [46] in America reveals the racial difference in the association between alcohol consumption and sleep quality. Their study pointed out that compared with white men and women, black men and women with moderate infrequent drinking were significantly less likely to report trouble falling and staying asleep. In our study, the subjects were likely to consume less alcohol due to poverty and its effect is not obvious. However, cultural factors should also be considered because Chinese women have a low rate of alcohol consumption. Thus, future studies must establish a cohort that will track the racial difference of relationships between alcohol consumption and sleep quality in Chinese people.

Meanwhile, in terms of the association between multiple chronic comorbid diseases and sleep quality, this study found that individuals with no more than one chronic disease had a lower risk of poor sleep due to poor physiological function [36], which was similar to previous reports [9]. We also found that illness during the past two weeks, which indicates poor health and quality of life, has significant effect on sleep quality of male and complete sample, that is similar to what was reported in previous study [13]. However, a strong positive correlation exists between quality of life and quality of sleep [8]. It is not clear why there is no link between illness during the past two weeks and sleep quality in female adults with a low income. No other relevant literature has been reported. This may be because illness during the past two weeks has a smaller impact on quality of life compared to males. We also observed a significant relationship between disability and poor sleep quality in both genders. After controlling for confounding factors, such as gender, sociodemographic and other health-related factors, disabled individuals were almost 44% more likely to report poor sleep quality than non-disabled individuals. This finding is similar to what Lobentanz et al. observed among multiple sclerosis patients [47]. Thus, disability status has an impact on physical and psychological domains of life quality.

Participants who live alone were not found to have an increased risk of poor sleep quality. No association was found in the logistic regression analysis after sex discrimination. This result was consistent with one previous study by Lo et al. [26], as they did not establish any statistically significant association between sleep quality and living alone. We think the possible reason is that although living alone can mean sleeping in a quieter environment, it can also keep negative emotions and stress from being releasing. Moreover, we found that a primarily vegetarian and balanced diet improved the sleep quality in males, but not in females. Stonge et al. [48] confirmed that higher saturated fat and lower fiber intake might lead to less slow-wave sleep and more night-time activity, which may reduce overall sleep quality. In this study, the assessment of diet was based on self-reports rather than quantitative measurements, which would be influenced by individual subjective factors. Therefore, there are certain differences in the effects of each diet on sleep quality. In future studies, quantitative methods can be used to accurately assess the association between dietary content of food and sleep quality for different genders. We found no association of salt intake and oil consumption with sleep quality in both genders. We think that these two indicators may have some potential relationship with sleep quality or some other factors are likely involved in the possible correlation between salt intake and sleep quality, which needs to be further studied.

Low-income adults are a vulnerable group in China and thus, studying their sleep quality is important. Our research has several strengths. First, our data are reliable because they are obtained from face-to-face interviews by investigators and not via telephone interviews. Second, our questionnaire (CPSQI) is comprehensive and reliable.

Nevertheless, we acknowledge that this study has several limitations. First, the study only involved Ezhou City. Thus, it is unlikely that these results can be generalized to all the low-income adults in rural areas of China. Second, we did not include other variables that might have an impact on sleep quality, such as body mass index, blood pressure, depression, number of young children and anxiety. These factors will be included in our future detailed investigations. Third, our study population is not sufficiently representative of the general population because the proportion of low-income older adults in rural areas in our sample is higher than it actually is and the education level is probably lower than that of ordinary low-income adults in China. More educated people and young people who migrated to the cities to earn a living were not included in the sample. Fourth, the data collection of some indicators in this study may not be accurate enough because of using a self-reported approach to collect data. Although PSQI has been widely used to assess the sleep quality, studies have pointed out the limitations of using such a scale in assessing the sleep quality [49]. There are also statistics that ignore the differences between adults and children, such as the intake of salt and edible oil. Therefore, the generalizability of our findings is limited.

## 5. Conclusions

Our study indicated that approximately 57% of low-income adults in rural areas experience poor sleep quality. The results suggested that sociodemographic, lifestyle and health-related factors are independently associated with poor sleep quality. Thus, comprehensive measures are necessary to improve sleep quality among low-income adults in rural areas. For example, educating them on the importance of a balanced diet and ways to reduce illness is reasonable and feasible. They can also be encouraged to appeal to local governments for help in improving their economic status. Furthermore, future studies with a large sample population that is sufficiently representative and with a larger number of variables can help to determine other related factors that affect sleep quality.

## Figures and Tables

**Table 1:** Participant characteristics by sleep quality.

Variable	Total	Good (CPSQI Score ≤ 5) *n* (%)	Poor (CPSQI Score > 5) *n* (%)	χ^2^	*p*-Value
**Gender**				43.99	0.000
Male	4120 (59.7)	1918 (64.2)	2202 (56.2)		
Female	2785 (40.3)	1072 (35.8)	1713 (43.8)		
**Age category, years**				151.06	0.000
18–39	727 (10.5)	436 (14.6)	291 (7.4)		
40–59	2570 (37.2)	1210 (40.5)	1360 (34.8)		
≥60	3608 (52.3)	1344 (44.9)	2264 (57.8)		
**Marital status**				54.31	0.000
Unmarried	1076 (15.6)	557 (18.6)	519 (13.3)		
Married	4135 (59.9)	1797 (60.1)	2338 (59.7)		
Divorced /widow/ widower	1694 (24.5)	636 (21.3)	1058 (27.0)		
**Employment**				75.81	0.000
Employed	828 (12.0)	475 (15.9)	353 (9.0)		
Unemployed	6077 (88.0)	2515 (84.1)	3562 (91.0)		
**Average annual income per person of a household (RMB)**				2631.38	0.000
≤1000	3587 (51.9)	498 (16.7)	3089 (78.9)		
1001–2300	3318 (48.1)	2492 (83.3)	826 (21.1)		
**Smoking status**				9.81	0.002
Yes	1467 (21.2)	688 (23.0)	779 (20.0)		
No	5438 (78.8)	2302 (77.0)	3136 (80.0)		
**Current alcohol drinker**				2.12	0.145
Yes	959 (13.9)	436 (14.6)	523 (13.4)		
No	5946 (86.1)	2554 (85.4)	3392 (86.6)		
**Educational level**				97.02	0.000
Illiterate	2356 (35.8)	978 (32.7)	1491 (38.1)		
Elementary school	2770 (40.1)	1221 (40.8)	1549 (39.6)		
Junior middle school	1356 (19.6)	656 (22.0)	700 (17.8)		
Senior middle school or higher	423 (4.5)	135 (4.5)	175 (4.5)		
**Salt intake (g/day)**				37.32	0.000
>6	2829 (41.0)	1323 (44.2)	1506 (38.4)		
>6 and ≤12	3494 (50.6)	1464 (49.0)	2030 (51.9)		
>12 and ≤18	528 (7.6)	191 (6.4)	337 (8.6)		
>18	54 (0.8)	12 (0.4)	42 (1.1)		
**Oil intake (g/day)**				27.64	0.000
≤25	2719 (39.4)	1282 (42.9)	1437 (36.7)		
>25 and ≤35	3608 (52.2)	1475 (49.3)	2133 (54.5)		
>35 and ≤45	473 (6.9)	194 (6.5)	279 (7.1)		
>45	105 (1.5)	39 (1.3)	66 (1.7)		
**Living arrangement**				27.35	0.000
Live alone	1571 (22.8)	590 (19.7)	981 (25.1)		
Live with others	5334 (77.2)	2400 (80.3)	2934 (74.9)		
**Dietary content of food**				68.21	0.000
Balanced diet	3240 (46.9)	1555 (52.0)	1685 (43.0)		
Primarily vegetarian	3579 (51.9)	1418 (47.4)	2161 (55.2)		
Primarily meat	86 (1.2)	17 (0.6)	69 (1.8)		
**Illness within 2 weeks**				9.13	0.003
No	3136 (45.4)	1296 (43.3)	1840 (47.0)		
Yes	3769 (54.6)	1694 (56.7)	2075 (53.0)		
**Self-reported disability**				19.94	0.000
Yes	2065 (29.9)	810 (27.1)	1255 (32.1)		
No	4840 (70.1)	2180 (72.9)	2660 (67.9)		
**Number of chronic diseases**				102.86	0.000
0	4351 (63.0)	2007 (67.1)	2344 (59.9)		
1	1271 (18.4)	584 (19.5)	687 (17.5)		
2	877 (12.7)	295 (9.96)	582 (14.9)		
≥3	406 (5.9)	104 (3.5)	302 (7.7)		

The Pittsburgh Sleep Quality Index score consists of 7 parts: subjective quality, latency, duration, habitual efficiency, disturbances, use of medications and daytime dysfunction. The CPSQI score is in the range of 0–21 from better to worse. Good sleep quality: CPSQI score ≤5; Poor sleep quality: CPSQI score >5. *n*: number; and %: the percentage of subjects. Chronic diseases: hypertension, diabetes, migraine, asthma, thyroid disease, heart disease, thrombosis, bronchitis/emphysema, osteoporosis, arthritis, cancer, stomach/peptic ulcer, cerebrovascular disease and other major physical diseases.

**Table 2:** Characteristics affecting quality of sleep by gender.

Variable, *n* (%)	Female		χ^2^	*p*	Male		χ^2^	*p*
	Good (CPSQI Score ≤ 5)	Poor (CPSQI Score > 5)			Good (CPSQI Score ≤ 5)	Poor (CPSQI Score > 5)		
**Age category, years**			90.74	0.000			68.65	0.000
18–39	160 (14.9)	98 (5.8)			276 (14.4)	192 (8.7)		
40–59	449 (41.9)	626 (36.5)			761 (39.7)	734 (33.3)		
≥60	463 (43.2)	988 (57.7)			881 (45.9)	1276 (58.0)		
**Marital status**			36.39	0.000			10.25	0.006
Unmarried	82 (7.7)	65 (3.8)			475 (24.8)	454 (20.6)		
Married	631 (58.9)	916 (53.5)			1166 (60.8)	1422 (64.6)		
Divorced/widow/widower	359 (33.4)	732 (42.7)			277 (14.4)	326 (14.8)		
**Employment**			30.11	0.000			38.22	0.000
Employed	941 (87.8)	1606 (93.8)			1574 (82.1)	1596 (88.8)		
Unemployed	131 (12.2)	107 (6.2)			344 (17.9)	246 (11.2)		
**Average annual income per person of a household (RMB)**			966.87	0.000			1656.05	0.000
≤1000	190 (17.7)	1336 (22.0)			308 (16.1)	1753 (79.6)		
1001–2300	882 (82.3)	377 (78.0)			1610 (83.9)	449 (20.4)		
**Smoking status**			2.10	0.147			0.61	0.436
Yes	9 (0.8)	25 (1.5)			679 (14.4)	754 (8.7)		
No	1063 (99.2)	1688 (98.5)			1239 (39.7)	1448 (33.3)		
**Current alcohol drinker**								
Yes	14 (1.3)	30 (1.8)	0.84	0.359	422 (22.0)	493 (22.4)	0.09	0.766
No	1058 (98.7)	1683 (98.2)			1496 (78.0)	1709 (77.6)		
**Educational level**			66.69	0.000			20.88	0.000
Illiterate	446 (41.6)	930 (54.3)			419 (21.8)	561 (25.5)		
Elementary school	387 (36.1)	553 (32.3)			834 (43.5)	996 (45.2)		
Junior middle school	160 (14.9)	185 (10.8)			496 (25.9)	515 (23.4)		
Senior middle school or higher	79 (7.4)	45 (2.6)			169 (8.8)	130 (5.9)		
**Salt intake (g/day)**			13.69	0.003			31.21	0.000
≤6	463 (43.2)	670 (39.1)			860 (44.8)	836 (38.0)		
>6 and ≤12	552 (51.5)	892 (52.1)			912 (47.6)	1138 (51.7)		
>12 and ≤18	51 (4.8)	138 (8.1)			140 (7.3)	199 (9.0)		
>18	6 (0.5)	13 (0.7)			6 (0.3)	29 (1.3)		
**Oil intake (g/day)**			8.34	0.039			21.95	0.000
≤25	464 (43.3)	651 (38.0)			818 (42.7)	786 (35.7)		
>25 and ≤35	537 (50.1)	923 (53.9)			938 (48.9)	1210 (54.9)		
>35 and ≤45	61 (5.7)	119 (6.9)			133 (6.9)	160 (7.3)		
>45	10 (0.9)	20 (1.2)			29 (1.5)	46 (2.1)		
**Living arrangement**			33.04	0.000			4.77	0.029
Live alone	179 (16.7)	446 (26.0)			411 (21.4)	535 (24.3)		
Live with others	893 (83.3)	1267 (74.0)			1507 (78.6)	1667 (75.7)		
**Dietary content of food**			27.29	0.000			37.82	0.000
Balanced diet	534 (49.8)	690 (40.3)			1021 (53.2)	995 (45.2)		
Primarily vegetarian	530 (49.4)	993 (58)			888 (46.3)	1168 (53.0)		
Primarily meat	8 (0.8)	30 (1.7)			9 (0.5)	39 (1.8)		
**Illness within 2 weeks**			4.36	0.037			3.60	0.058
No	588 (54.9)	870 (50.8)			1106 (57.7)	1205 (54.7)		
Yes	484 (45.1)	843 (49.2)			812 (42.3)	997 (435.)		
**Self-reported disability**			18.40	0.000			8.89	0.003
Yes	230 (21.5)	493 (28.8)			580 (30.2)	762 (34.6)		
No	842 (78.5)	1220 (71.2)			1338 (69.8)	1440 (65.4)		
**Number of chronic diseases**			39.69	0.000			58.04	0.000
0	697 (65.0)	992 (57.9)			1310 (68.3)	1352 (61.4)		
1	210 (19.6)	299 (17.5)			374 (19.5)	388 (17.6)		
2	122 (11.4)	264 (15.4)			173 (9.0)	318 (14.4)		
≥3	43 (4.0)	158 (9.2)			61 (3.2)	144 (6.5)		

The Pittsburgh Sleep Quality Index score consists of 7 parts: subjective quality, latency, duration, habitual efficiency, disturbances, use of medications and daytime dysfunction. The CPSQI score is in the range of 0–21 from better to worse. Good sleep quality: CPSQI score ≤ 5; Poor sleep quality: CPSQI score >5. *n*: number; and %: the percentage of subjects. Chronic diseases: hypertension, diabetes, migraine, asthma, thyroid disease, heart disease, thrombosis, bronchitis/emphysema, osteoporosis, arthritis, cancer, stomach/peptic ulcer, cerebrovascular disease and other major physical diseases.

**Table 3:** Gender and age category scores of PSQI component scores and total score in all participants.

Variables	Overall (*n* = 6905)	Male (*n* = 4120)	Female (*n* = 2785)	t	*p*	18–39 Years (*n* = 727)	40–59 Years (*n* = 2570)	≥60 Years (*n* = 3608)	F	*p*
Self-rated sleep quality, M ± SD	1.29 ± 0.01	1.24 ± 0.01	1.38 ± 0.02	7.30	0.000	1.04 ± 0.75	1.26 ± 0.79	1.37 ± 0.78	59.83	0.000
Sleep latency, M ± SD	1.28 ± 0.01	1.22 ± 0.01	1.37 ± 0.02	7.17	0.000	1.14 ± 0.79	1.24 ± 0.82	1.34 ± 0.83	24.68	0.000
Sleep duration, M ± SD	0.77 ± 0.01	0.76 ± 0.01	0.77 ± 0.02	0.68	0.50	0.64 ± 0.81	0.73 ± 0.81	0.82 ± 0.81	18.72	0.000
Sleep efficiency, M ± SD	0.16 ± 0.01	0.13 ± 0.01	0.19 ± 0.01	4.90	0.000	0.11 ± 0.40	0.13 ± 0.43	0.18 ± 0.49	11.52	0.000
Sleep disturbance, M ± SD	1.42 ± 0.01	1.38 ± 0.01	1.48 ± 0.01	5.15	0.000	1.12 ± 0.71	1.38 ± 0.72	1.54 ± 0.74	120.29	0.000
Need for sleep medications, M ± SD	0.20 ± 0.01	0.19 ± 0.01	0.22 ± 0.01	1.64	0.10	0.42 ± 0.93	0.21 ± 0.63	0.15 ± 0.49	59.84	0.000
Daytime dysfunction, M ± SD	1.27 ± 0.01	1.24 ± 0.01	1.31 ± 0.02	3.20	0.0014	1.06 ± 0.96	1.20 ± 0.93	1.35 ± 0.93	40.99	0.000
PSQI total score, M ± SD	6.39 ± 2.99	6.17 ± 0.04	6.72 ± 0.06	7.47	0.000	5.53 ± 3.17	6.12 ± 2.96	6.76 ± 2.91	69.89	0.000

M: mean; SD: standard deviation; PSQI: Pittsburgh Sleep Quality Index.

**Table 4:** Multivariable logistic regression of factors associated with poor sleep quality.

Independent Variables	Unadjusted OR (95% CI)	*p*	Adjusted OR_total_^a^ (95% CI)	*p*
**Gender (male as reference)**				
Female	1.39 (1.26, 1.53)	0.000	1.26 (1.08, 1.47)	0.003
**Age category, years (≥60 as reference)**				
18–39	0.40 (0.34, 0.47)	0.000	0.46 (0.36, 0.59)	0.000
40–59	0.67 (0.60, 0.74)	0.000	0.86 (0.74, 1.00)	0.064
**Marital status (“divorced /widow/ widower” as reference)**				
Married	0.56 (0.48, 0.65)	0.000	0.71 (0.56, 0.89)	0.004
Unmarried	0.78 (0.70, 0.88)	0.000	0.99 (0.84, 1.17)	0.935
**Employment (unemployed as reference)**				
Employed	0.52 (0.45, 0.61)	0.000	0.49 (0.41, 0.60)	0.000
**Average annual income per person of a household (RMB) (≤1000 as reference)**				
1001–2300	0.05 (0.04, 0.06)	0.000	0.05 (0.04, 0.06)	0.000
**Educational level (“Senior middle school or higher” as reference)**				
Illiterate	2.44 (1.98, 3.02)	0.000	1.51 (1.23, 2.14)	0.004
Elementary school	1.80 (1.46, 2.21)	0.000	1.45 (1.12, 1.92)	0.007
Junior middle school	1.51 (1.21, 1.89)	0.000	1.39 (1.02, 1.81)	0.026
**Salt intake (g/day) (>18 as reference)**				
≤6	0.33 (0.17, 0.62)	0.001	0.58 (0.24, 1.39)	0.220
>6 and ≤12	0.40 (0.21, 0.76)	0.005	0.63 (0.27, 1.46)	0.279
>12 and ≤18	0.50 (0.26, 0.98)	0.044	0.76 (0.32, 1.79)	0.532
**Oil intake(g/day) (>45 as reference)**				
≤25	0.66 (0.44, 0.99)	0.045	0.49 (0.27, 0.86)	0.014
>25 and ≤35	0.85 (0.57, 1.28)	0.443	0.59 (0.34, 1.01)	0.054
>35 and ≤45	0.85 (0.55, 1.31)	0.465	0.60 (0.34, 1.05)	0.076
**Living arrangement (live alone as reference)**				
Live with others	0.74 (0.66, 0.83)	0.000	0.90 (0.75, 1.08)	0.276
**Dietary content of food (primarily meat as reference)**				
Balanced diet	0.27 (0.16, 0.46)	0.000	0.26 (0.14, 0.50)	0.000
Primarily vegetarian	0.38 (0.22, 0.64)	0.000	0.29 (0.15, 0.55)	0.000
**Illness within 2 weeks (Yes as reference)**				
No	1.16 (1.05, 1.28)	0.003	0.78 (0.63, 0.97)	0.027
**Total number of chronic diseases (≥3 as reference)**				
0	0.40 (0.32, 0.51)	0.000	0.40 (0.29, 0.57)	0.000
1	0.41 (0.32, 0.52)	0.000	0.55 (0.40, 0.75)	0.000
2	0.68 (0.52, 0.88)	0.004	0.75 (0.54, 1.05)	0.095
**Self-reported disability (Yes as reference)**				
No	0.79 (0.71, 0.87)	0.000	0.56 (0.48, 0.65)	0.000

^a^ Adjusted for gender, age category, marital status, employment, average household income, living arrangement, educational level, salt intake, oil intake, dietary content of food, illness during the 2 weeks, total number of chronic diseases and self-reported disability. OR: odds ratio; and CI: confidence interval. Chronic diseases: hypertension, diabetes, migraine, asthma, thyroid disease, heart disease, thrombosis, bronchitis/emphysema, osteoporosis, arthritis, cancer, peptic ulcer, cerebrovascular disease and other major physical diseases.

**Table 5:** Adjusted associated factors of poor sleep quality by gender.

Independent Variables	Female		Male	
	OR ^b^ (95% CI)	*p*	OR ^b^ (95% CI)	*p*
**Age category, years (≥60 as reference)**				
18–39	0.41 (0.27, 0.61)	0.000	0.51 (0.37, 0.71)	0.000
40–59	0.96 (0.74, 1.24)	0.736	0.83 (0.68, 1.01)	0.064
**Marital status (“divorced /widow/ widower” as reference)**				
Married	0.51 (0.31, 0.84)	0.008	0.89 (0.66, 1.19)	0.419
Unmarried	0.84 (0.66, 1.05)	0.128	1.22 (0.95, 1.57)	0.124
**Employment (unemployed as reference)**				
Employed	0.48 (0.33, 0.68)	0.000	0.49 (0.39, 0.63)	0.000
**Average annual income per person of a household (RMB) (≤1000 as reference)**				
1001–2300	0.06 (0.05, 0.07)	0.000	0.05 (0.04, 0.06)	0.000
**Living arrangement (live alone as reference)**				
Live with others	0.89 (0.68, 1.18)	0.430	0.89 (0.70, 1.14)	0.351
**Educational level (“Senior middle school or higher” as reference)**				
Illiterate	2.15 (1.32, 3.52)	0.002	1.19 (0.84, 1.70)	0.325
Elementary school	1.93 (1.18, 3.16)	0.009	1.25 (0.91, 1.74)	0.174
Junior middle school	1.65 (0.96, 2.83)	0.071	1.26 (0.90, 1.77)	0.185
**Salt intake (g/day) (>18 as reference)**				
≤6	1.29 (0.34, 4.87)	0.709	0.35 (0.11, 1.12)	0.077
>6 and ≤12	1.30 (0.36, 4.71)	0.690	0.39 (0.12, 1.22)	0.105
>12 and≤18	1.93 (0.52, 7.18)	0.328	0.42 (0.13, 1.32)	0.139
**Oil intake (g/day) (>45 as reference)**				
≤25	0.42 (0.15, 1.18)	0.098	0.52 (0.26, 1.05)	0.068
>25 and ≤35	0.51 (0.19, 1.39)	0.189	0.62 (0.32, 1.21)	0.159
>35 and ≤45	0.54 (0.19, 1.51)	0.241	0.62 (0.31, 1.23)	0.169
**Dietary content of food (primarily meat as reference)**				
Balanced diet	0.43 (0.16, 1.12)	0.083	0.17 (0.07, 0.41)	0.000
Primarily vegetarian	0.49 (0.19, 1.28)	0.148	0.19 (0.08, 0.45)	0.000
**Illness during the last 2 weeks (Yes as reference)**				
No	0.92 (0.65, 1.29)	0.617	0.69 (0.52, 0.91)	0.009
**Total number of chronic diseases (≥3 as reference)**				
0	0.46 (0.27, 0.78)	0.004	0.37 (0.23, 0.60)	0.000
1	0.57 (0.36, 0.91)	0.019	0.54 (0.35, 0.84)	0.006
2	0.64 (0.39, 1.04)	0.069	0.88 (0.55, 1.40)	0.589
**Self-reported disability (Yes as reference)**				
No	0.45 (0.35, 0.57)	0.000	0.63 (0.53, 0.76)	0.000

^b^ Adjusted for age category, marital status, employment, average household income, living arrangement, educational level, salt intake, oil intake, dietary content of food, illness during the 2 weeks, total number of chronic diseases and self-reported disability. OR: odds ratio; and CI: confidence interval. Chronic diseases: hypertension, diabetes, migraine, asthma, thyroid disease, heart disease, thrombosis, bronchitis/emphysema, osteoporosis, arthritis, cancer, peptic ulcer, cerebrovascular disease and other major physical diseases.

## Abbreviations

The following abbreviations are used in this manuscript:	
CI	Confidence interval
CPSQI	Chinese version Pittsburgh Sleep Quality Index
FFQ	Food Frequency Questionnaire
OR	Odds ratio
PSQI	Pittsburgh Sleep Quality Index
SD	Standard deviation
